# Bayesian clinical reasoning in the first opinion approach to a dog with suspected thoracolumbar pain

**DOI:** 10.1111/jsap.13528

**Published:** 2022-06-25

**Authors:** S. Khan, P. Freeman

**Affiliations:** ^1^ Queen's Veterinary School Hospital Cambridge CB3 0ES UK

## Abstract

Whilst multi‐planar imaging has allowed advances in diagnosis and treatment of canine spinal cord disorders, it is sometimes inaccessible to pet owners leading to a reliance on imaging modalities and ancillary tests that are more readily available. For this reason, this essay considers how Bayesian clinical reasoning may aid in deciding which tests, if any, are most useful for the diagnosis of spinal disease in clinical practice and choosing reasonable empiric therapies.

## INTRODUCTION

Spinal disorders are a common reason for presentation in clinical practice and these cases present a significant challenge to the practitioner with more than 40 potential differential diagnoses to consider (da Costa & Moore 2010, Parent 2010). Previous studies have shown that history, physical examination and neurological examination findings can aid in producing an accurate and prioritised list of differential diagnoses (Parent 2010, Cardy *et al*. 2015), however, multi‐planar imaging is often required for further differentiation, with MRI and CT being considered the most valuable (da Costa & Samii 2010). Whilst such imaging has allowed advances in diagnosis and treatment of canine spinal cord disorders, it is often inaccessible to pet owners for financial, geographical or, more recently, global pandemic reasons. This leads to a reliance on imaging modalities and ancillary tests that are more readily available such as survey radiography or in‐house blood analysis. For this reason, we wished to consider how Bayesian clinical reasoning may aid in deciding if any sort of investigation is really appropriate in such a situation, or if in fact general practitioners may be better advised to consider the likely differential diagnoses based rather on history and signalment alone and use this information to decide on reasonable empiric treatments. To illustrate this approach, we looked at the relatively common example of a dog with acute apparent thoracolumbar back pain which persists or recurs over days to a few weeks despite empirical treatment, but without developing additional neurological deficits or other clinical signs.

## WHAT IS BAYESIAN CLINICAL REASONING?

Bayesian clinical reasoning is a way of taking into account the known pretest probability of a disorder (based on a mix of published reviews, studies and case reports, and expert opinion), and using this information to interpret the probability of that disorder following the results of a diagnostic test. In other words, it involves using our prioritised differential diagnosis list to inform and interpret our use of the diagnostic investigations we undertake. This is something we all do unconsciously every day, prioritising our differential diagnoses based on our previous experience and knowledge. That is to say that we are unconsciously setting the pretest probability of a disease in our everyday clinical practice. If we use the collective experience of the profession when presented with a dog with back pain, we can not only prioritise differential diagnoses but also investigative tests and make more efficient strides to a diagnosis even when multi‐planar imaging and referral is unavailable.

## CLINICAL SETTING

In cases presenting with suspected thoracolumbar pain, it can sometimes be challenging to differentiate abdominal pain from true spinal pain. In the absence of other clinical (neurological) signs that would help to localise the pain, both neurological and non‐neurological diagnoses should be considered. In this instance a thorough history and physical examination can help rule out traumatic disorders such as fractures, luxations or penetrating wounds and, a majority of causes of acute abdominal pain originating in the gastrointestinal, urogenital and hepatobiliary systems. Acute pancreatitis is known to present with vague, non‐specific signs (Berman *et al*. 2020) including pain alone, and of the numerous causes of cranial abdominal pain, it is the authors' experience that pancreatitis is the most common misdiagnosis in cases of intervertebral disc extrusion (IVDE), and vice versa. Therefore, whilst not the only one, we would consider this to be the most common non‐neurological differential in this scenario. The most common differential diagnoses to be considered for true thoracolumbar pain are IVDE, discospondylitis, neoplasia (of the vertebrae, meninges and spinal cord), and sterile meningomyelitis (MUO) (Cardy *et al*. 2015, Dewey & da Costa 2016a). Whilst many other causes have been reported for thoracolumbar pain, these are extremely rare and generally limited to isolated case reports. Our purpose is to aid in the diagnosis of those conditions that are either common or require early diagnosis in order to avoid inappropriate management. So, what is the most appropriate next step when presented with a dog with suspected thoracolumbar pain, but when logistical or financial constraints are an impediment to referral for a complete diagnostic investigation? In the next section we will review a series of readily available diagnostic investigations which may be considered in such a case.

At this juncture, it is also important to consider the value of the natural progression of the diseases we have considered and the likely signalment of such patients. In many ways Bayesian clinical reasoning could almost consider clinical progression a form of test but since accurate data for prevalence are lacking, we will reserve discussion of clinical progression until after consideration of all tests. It is also true that in many scenarios external pressures force clinicians to undertake some form of investigation to justify their decision making and so a “wait and see approach” is as unavailable as referral and the effect of this pressure should not be discounted.

### Survey radiography

Survey radiography is a readily available diagnostic procedure in a majority of first opinion practices and can be used to assess for signs of intervertebral disc disease, vertebral neoplasia and discospondylitis. Abdominal radiography has also been investigated as a diagnostic tool for pancreatitis but was reported to have a very low sensitivity and specificity so will not be discussed further (Hess *et al*. 1998). When radiographing the spine, it is important to remember that due to the divergence of the x‐ray beam the spine should be imaged in short segments and sedation or general anaesthesia should be used to facilitate correct positioning (Olby & Thrall 2014). Failure to employ good radiographic techniques and to assess the images as a whole, as with most diagnostic procedures, is likely to lead to false negatives or false positives, and therefore invalidate results. For the purposes of this article, we will assume radiography is performed appropriately so that findings may be interpreted with confidence.

#### Intervertebral disc herniation

Previous studies investigating the value of survey radiography in the diagnosis of intervertebral disc disease have assessed several different signs for diagnostic utility (Lamb *et al*. 2002, Abdel‐Hakiem *et al*. 2015). Mineralisation of intervertebral discs has been shown to be a useful predictor of future IVDE and a marker of intervertebral disc degeneration in dachshunds (Jensen *et al*. 2008, Rohdin *et al*. 2010, Lappalainen *et al*. 2014). Whilst this may mean radiography is a useful technique for screening dogs prior to breeding, with schemes operating in several countries, it does not aid in the diagnosis of acute disc extrusion (Stigen *et al*. 2019). Narrowing of the intervertebral disc space, presence of mineralised disc material within the vertebral canal and vacuum phenomenon have also been investigated (Lamb *et al*. 2002). Whilst narrowing of the intervertebral disc space was found to be the most valuable diagnostic sign in cases of IVDH it had a sensitivity of just 64% to 69% (Lamb *et al*. 2002). Other studies have reported that survey radiography correctly identified between 68% and 72% of cases of canine IVDH (Kirberger *et al*. 1992, Olby *et al*. 1994, Brisson 2010).

Unfortunately, previous reports do not provide reliable estimates of the specificity or predictive value of survey radiography meaning that the power of a normal radiograph to rule out a disc extrusion has not been interrogated. It is, however, the authors' experience that narrowing of the intervertebral disc space is in fact a reasonably reliable predictor of extrusion and the inverse is true of a normal radiograph.

#### Discospondylitis

Radiography is often used as a screening tool for discospondylitis. Signs include vertebral end plate osteolysis and collapse or widening of the intervertebral disc space (Ruoff *et al*. 2018). A separate radiographic diagnosis of physitis has also been described in dogs less than two years of age affecting the caudal physeal region of the vertebral body as opposed to the end plates. Unfortunately, radiographic signs often lag behind clinical infection (sometimes by up to 2 to 4 weeks) making it impossible to rule out discospondylitis on the basis of a normal spinal radiograph (Olby & Thrall 2014). Therefore a misdiagnosis of IVDH could be possible if a narrowed disc space were the solitary radiographic finding or radiographic signs consistent with discospondylitis not be visible, especially early on in the disease process or in younger dogs (Burkert *et al*. 2005, Ruoff *et al*. 2018). In this situation it is likely that the management strategy for the individual animal would not eliminate the pain associated with such an infection due to lack of antibiotic therapy, and therefore further investigations, perhaps including repeating radiography, may be considered.

#### Vertebral neoplasia

Common tumours affecting the vertebral body include osteosarcoma, chondrosarcoma, myeloma and fibrosarcoma (LeCouter & Withrow 2007, da Costa 2008). Studies exploring the use of survey radiography in patients with tumours that affect the vertebrae found bone lysis, bone proliferation and pathological fracture in the body or vertebral lamina to be common findings (Petersen *et al*. 2008, Valentim *et al*. 2018). One such study which aimed to assess various different imaging modalities for their ability to detect vertebral neoplasia found that in cases whose end diagnosis was one of vertebral neoplasia, signs were evident on survey radiography in 61% of the cases (Valentim *et al*. 2018). We did not aim to identify alternative diagnoses when evaluating the spinal radiographs, and therefore cannot comment on the likelihood of misdiagnosis of vertebral body tumour as IVDH based on survey radiographs from our study.

### Canine pancreatic lipase

Increased serum canine pancreatic lipase (cPLI) concentrations are considered to be a sensitive and specific test for canine acute pancreatitis (Neilson‐Carley *et al*. 2011, Trivedi *et al*. 2011) but increased serum cPLI concentrations have also been reported in non‐pancreatic disease (Steiner *et al*. 2003a, 2003b, Chartier *et al*. 2014). It is most important to consider whether an elevated cPLI would be capable of definitively differentiating acute pancreatitis from intervertebral disc disease as these are potentially two common conditions which may present similarly (acute poorly localisable pain from thoracolumbar spine or cranial abdomen) and require differentiation because they have significantly different management regimes, prognosis and client communication requirements. It has been demonstrated that a proportion of dogs with histological evidence of acute pancreatitis may have a normal cPLI (Trivedi *et al*. 2011) and, cPLI can also be significantly elevated in dogs with intervertebral disc herniation (Schueler *et al*. 2018). In this population of dogs, the elevated cPLI had no association with gastrointestinal signs, neuroanatomical localisation or administration of corticosteroids or non‐steroidal anti‐inflammatory drugs. Whilst accurate data allowing interpretation of cPLi is lacking, it is clear that further studies are required to evaluate the relationship between IVDH and cPLI and until such studies are undertaken and published, care should be taken in ruling out IVDH on the basis of an elevated cPLI in cases where pain is the sole presenting sign.

### C‐reactive protein

C‐reactive protein (CRP) is an acute phase protein produced in the canine liver. Serum concentrations are low in healthy dogs, but large increases occur in acute inflammation. CRP has been evaluated in many canine diseases including neurological diseases. In one such study there was no significant increase in serum CRP in intervertebral disc disease, necrotising meningoencephalitis or tumours of the CNS (Nakamura *et al*. 2008). Only 63% of cases of discospondylitis in dogs may have a having a detectable increase in CRP (Bush *et al*. 2016, Nye *et al*. 2020). By comparison CRP has been demonstrated to be elevated in canine acute pancreatitis (Nakamura *et al*. 2008), therefore, providing a potential avenue for differentiating acute pancreatitis from IVDH (perhaps more useful than cPLI as discussed above). It is, however, not established that all cases of intervertebral disc herniation have a normal CRP (Nakamura *et al*. 2008) and, if elevated, discospondylitis cannot be ruled out without additional tests.

## THE BAYESIAN APPROACH

So far, we have discussed some ancillary tests that are readily available in general practice and could provide a pathway for a tentative diagnosis in the case of a dog with possible thoracolumbar pain and minimal or no neurological deficits. How does Bayesian clinical reasoning (as introduced earlier) affect our approach to the potential usefulness of these diagnostic tests? As stated above, Bayesian clinical reasoning involves using the pretest probability of an outcome (in this case a diagnosis) to inform the posttest probability. This means we must first consider the prevalence of each possible differential diagnosis in our patients. In order to estimate this, it is important to take into account signalment, including particularly breed predisposition. For example, the middle‐aged chondrodystrophic dog is far more likely to be suffering from an IVDE than any other possible cause (Parent 2010). More generally IVDE and acute pancreatitis should be considered the most common differentials (da Costa & Moore 2010, da Costa & Samii 2010) with discospondylitis being uncommon in the United Kingdom and more commonly affecting the L7‐S1 disc space (Thomas 2000, Burkert *et al*. 2005), and vertebral and spinal neoplasia generally affecting older patients and again being much less common (Cardy *et al*. 2015, Dewey & da Costa 2016b).

We should also consider at this point the characteristics of a test that would make it valuable in the context of Bayesian clinical reasoning. Our aim is to either strongly increase the suspicion of a rare disease, or alternatively, to definitively rule out a common disease (with the latter probably being more important). To put this more simply, it is questionable in our opinion whether we should, as vets, be using limited financial resources to rule in common diseases or rule out uncommon ones. Therefore, when testing for a rare disease the specificity and positive predictive values are most important to consider, and when testing for a common disease the sensitivity and negative predictive values are key. We will now consider each of the aforementioned tests in the context of Bayesian reasoning.

It is important to note that much of the data required for a truly informed Bayesian approach is unavailable, and therefore the authors have instead used their own and others' clinical experience to make a judgment on the value of each investigation, without a defined cut‐off of sensitivity or specificity. For further information on sensitivity, specificity, odds ratios and likelihood ratios and their use in clinical reasoning the reader is directed to McKenzie (2021).

## SERUM CRP

As already discussed, serum CRP is an acute phase protein which is elevated in acute inflammation, and some of our differentials involve inflammatory processes. To be useful in our scenario an increased CRP would have to significantly increase the likelihood of a rare condition such as discospondylitis or definitively exclude a common condition like IVDE. Ideally it would also differentiate between two inflammatory conditions such as pancreatitis and discospondylitis in order to avoid a scenario whereby it simply justifies more testing, but we know that CRP elevations are unable to differentiate between different causes of acute inflammation (Nakamura *et al*. 2008). Specificity data is lacking for CRP and discospondylitis (Bush *et al*. 2016, Nye *et al*. 2020), but we know that a significant proportion of cases may have CRP within normal limits. CRP elevation is also unlikely to be sufficient to completely exclude IVDE, since some cases have been shown to have increased CRP (Nakamura *et al*. 2008). In the case of other rare inflammatory causes of thoracolumbar pain, the fact CRP has been demonstrated to be normal in necrotizing meningoencephalitis (Nakamura *et al*. 2008) means it is not a useful test in significantly increasing the likelihood of sterile inflammatory disease. We, therefore, judge CRP measurement to be of little value in this clinical scenario.

## SERUM CPLI

Serum cPLi is considered to be a sensitive and specific test for canine pancreatitis (Neilson‐Carley *et al*. 2011, Trivedi *et al*. 2011) but does it meet the requirements of a useful Bayesian test as previously laid out. In order to do this a normal cPLI would have to definitively rule out acute pancreatitis and an abnormal or increased cPLI would have to not only significantly increase the chances of a true diagnosis of acute pancreatitis, but also exclude a diagnosis of IVDE. The fact that a normal cPLI does not definitively rule out pancreatitis (Trivedi *et al*. 2011) and an increased cPLI has been demonstrated in a proportion of dogs with IVDE in the absence of gastrointestinal signs (Schueler *et al*. 2018) suggests it does not meet this standard. In addition, it has been postulated that thoracolumbar myelopathies may be able to induce acute pancreatitis in human patients (Carey *et al*. 1977), further confusing the interpretation of the test. Therefore again we would judge this test not useful in the absence of GI signs in this scenario.

## SURVEY RADIOGRAPHY

As discussed before survey radiography can be useful in the diagnosis of intervertebral disc disease, discospondylitis and neoplasia (Lamb *et al*. 2002, Ruoff *et al*. 2018, Valentim *et al*. 2018) whilst having little to no use in the diagnosis of pancreatitis (Hess *et al*. 1998). Given the prevalence of intervertebral disc disease perhaps the most useful consideration is either the negative predictive value or likelihood ratio for *not* having an IVDE when a narrowed disc space is not identified. Remember the usefulness of a test in the case of a common condition is in its ability to definitively rule out that condition. Unfortunately, such data is lacking, but it is the authors' experience that a dog with an extrusion will very commonly have a narrowed disc space. In other words, if we take a good quality spinal radiograph and do not identify a narrowed disc space, we have significantly reduced the chance of the diagnosis being IVDE. However, we have not completely excluded it!

To be useful for discospondylitis and neoplasia, survey radiography should be able to significantly increase their likelihood since these diagnoses are uncommon. In this instance a negative radiograph is of little use but a radiograph which demonstrates evidence of neoplasia or discospondylitis is very useful (it definitively rules in an uncommon disease). Whilst survey radiography does come closer to meeting the criteria set out by Bayesian reasoning, it is our experience that in most scenarios it would simply either increase our index of suspicion for the common condition of IVDE, which is already likely due to its prevalence, or rule out some uncommon diseases. Even here the less common conditions cannot be completely ruled out, since *discospondylitis* as already stated may take several weeks to produce radiographic changes (Olby & Thrall 2014) and may have a narrowed disc space as its solitary radiographic finding, and not all spinal neoplasia is visible radiographically. While this may seem problematic, it is rare for discospondylitis to progress to requiring emergency surgical intervention, and so repeating radiographs at a later date is a valid method of reducing the risk of misdiagnosis here.

## SUMMARY

It is clear that, based upon the available data, none of the tests described above meet the criteria set out by Bayesian reasoning. So, in the absence of a good test what is the most useful course of action in a dog with non‐specific thoracolumbar pain at their first presentation? Perhaps the most useful information in terms of arriving at a likely diagnosis will be the progression of the clinical signs with empirical therapy. A dog who progresses to gastrointestinal signs is inevitably more likely to have pancreatitis, whilst a dog who progresses to pelvic limb ataxia and proprioceptive deficits will usually have suffered an IVDE. It is also likely that with rest and appropriate analgesia, a significant proportion of individuals will simply improve, and a definitive diagnosis will not have proved necessary (also helping to exclude discospondylitis and neoplasia).

If we simultaneously consider the signalment we can refine this process even further. Whilst specific prevalence data for each breed and age are lacking, clinical experience tells us that the 5‐year‐old dachshund will most likely have a disc extrusion, whilst the middle‐aged miniature schnauzer more often has pancreatitis. In essence, clinical progression combined with signalment can almost be considered to be a “test” which will not only meet many of the criteria previously described but also, in the authors' experience, lead to many dogs recovering without the requirement for further testing.

The example of a dog whose pain persists or recurs, without deterioration, over days to weeks despite empirical therapy provides a subtly different dilemma. Whilst the relative probability of each differential is largely unchanged, neoplasia and discospondylitis should be considered a little more likely and pancreatitis a little less so. It is also likely that in this scenario external pressures mean a “wait and see approach” is no longer an option without some further justification. In fact, frustration over a lack of clinical improvement, despite no evidence of progression, is a common reason for referral or inappropriate changes to treatment. In this situation we must consider which of our readily available tests comes closest to meeting the Bayesian criteria laid out previously. The information and opinions expressed here suggest that survey spinal radiography is probably the most appropriate test to perform in such a scenario.

## CONCLUSION

In conclusion, Bayesian clinical reasoning is a form of clinical reasoning that we utilise unconsciously every day to prioritise differential diagnoses. The current lack of prevalence data as well as a requirement to use a certain amount of personal experience regarding signalment and clinical signs make this approach challenging. However hopefully by illustrating its use in this scenario of non‐specific thoracolumbar pain with no additional neurological deficits, we have shown how it can aid in determining which tests, if any, are most useful especially when significant financial constraints are in place. Based upon the requirements of Bayesian clinical reasoning and the existing evidence, our conclusion is that no test meets all the requirements for a perfect Bayesian test. Instead at first presentation the signalment and progression of clinical signs are likely to be the most useful pieces of information diagnostically, but should signs recur or fail to improve with empirical therapy, survey radiography comes closest to satisfying the requirements for a suitable empirical (and relatively inexpensive and widely available) test according to the tenets of Bayesian reasoning.

### Conflict of interest

None of the authors of this article has a financial or personal relationship with other people or organisations that could inappropriately influence or bias the content of the paper.

### Author contributions

**Sam Khan:** Conceptualization (equal); writing – original draft (lead); writing – review and editing (equal). **Paul Freeman:** Conceptualization (equal); methodology (equal); supervision (lead); writing – original draft (supporting); writing – review and editing (equal).

## Data Availability

As per the data sharing policy, data associated with this paper are available.
